# Age- and Sex-Specific Characteristics of Right Ventricular Compacted and Non-compacted Myocardium by Cardiac Magnetic Resonance

**DOI:** 10.3389/fcvm.2021.781393

**Published:** 2021-12-07

**Authors:** Anna Réka Kiss, Zsófia Gregor, Ádám Furák, Liliána Erzsébet Szabó, Zsófia Dohy, Béla Merkely, Hajnalka Vágó, Andrea Szűcs

**Affiliations:** Heart and Vascular Center of Semmelweis University, Budapest, Hungary

**Keywords:** right ventricle, cardiac magnetic resonance, trabeculated myocardium, compacted myocardium, age-related, sex-related, characteristics

## Abstract

The age and sex-specific characteristics of right ventricular compacted (RV-CMi) and RV-trabeculated myocardial mass (RV-TMi) and the determinants of RV myocardium are less well-studied; however, in different conditions, these might provide additional diagnostic information. We aimed to describe the age- and sex-specific characteristics of RV-CMi, RV-TMi, and RV volumetric and functional parameters and investigate the determinants of RV myocardial mass with cardiac magnetic resonance (CMR). Two hundred healthy Caucasian volunteers free of known cardiovascular or systemic diseases were prospectively enrolled in this study. Four different age groups were established with equal numbers of males and females: Group A (*n* = 50, 20-29 years, mean age: 24.3 ± 3.2 years), Group B (*n* = 50, 30-39 years, mean age: 33.6 ± 2.6 years), Group C (*n* = 50, 40-49 years, mean age: 44.7 ± 2.7 years), and Group D (*n* = 50, ≥50 years, mean age: 55.1 ± 3.9 years). Left ventricular (LV) and RV volumetric, functional, CMi, and TMi values were measured with a threshold-based post-processing CMR method. The volumetric parameters, RV-CMi, and RV-TMi values were larger, and the ejection fraction (EF) was lower in males. The RV-CMi did not correlate with age in either of the sexes, while the RV-TMi decreased with age in females but remained stable in males. The RV-TMi and RV-CMi correlated positively with RV volumetric parameters, the LV-CMi, the LV-TMi, and each other in both sexes. LV-TMi, LV-CMi, RV end-systolic volume, and sex were independent predictors of RV-TMi. Understanding the characteristics of RV-trabeculated and RV-compacted myocardium might have additive value in diagnosing different conditions with RV hypertrophy or hypertrabeculation.

## Introduction

The age- and sex-related characteristics of right ventricular (RV) volumetric and functional parameters have already been described in healthy populations (1, 2). In contrast, the characteristics of RV-compacted (RV-CMi) and RV-trabeculated myocardial mass (RV-TMi) and the quantification and determinants of RV myocardium are less well-studied. However, these might have diagnostic, differential diagnostic, and prognostic relevance in different conditions affecting the right heart (3–8). The segmentation used to measure RV myocardial mass is challenging, and the physiologic presence of the high number of endocardial trabeculae makes its quantification even more difficult. Threshold-based CMR post-processing software can accurately differentiate endocardial trabeculation and papillary muscles from the blood pool and measure the mass of both compacted and trabeculated myocardium (9). This study aimed to describe the age- and sex-specific characteristics of RV volumetric and functional parameters and RV-compacted and RV-trabeculated myocardial masses and investigate the relationship between RV trabeculation and RV volumetric and functional parameters and LV trabeculation using threshold-based CMR post-processing software.

## Materials and Methods

### Study Population

One hundred male and one hundred female Caucasian volunteers free of known diseases were prospectively enrolled between January 2019 and September 2020. Each participant completed a questionnaire including demographic data, cardiovascular symptoms, medical history, medication use, and sport activity. The inclusion criteria were as follows: the absence of cardiac diseases (congenital abnormality, ischemic heart disease, arrhythmia, valvular heart disease, cardiomyopathy), lack of sudden cardiac death in the family history, absence of other systemic disorders (hypertension-related, pulmonary, renal, gastrointestinal, metabolic, autoimmune, endocrine, psychiatric, oncological, neuromuscular, or other hereditary diseases), and sport activity <6 h per week (10). CMR examination without an injection of contrast agent, blood pressure measurement, 12-lead resting electrocardiography, and echocardiography were performed on the same day, and no abnormalities were found.

The study population was divided into four different age groups, which were set up with equal numbers of males and females: Group A (*n* = 50, 20-29 years, mean age: 24.3 ± 3.2 years), Group B (*n* = 50, 30-39 years, mean age: 33.6 ± 2.6 years), Group C (*n* = 50, 40-49 years, mean age: 44.7 ± 2.7 years), and Group D (*n* = 50, 50-66 years, mean age: 55.1 ± 3.9 years). The male and female participants' baseline demographic, morphometric, and left and right ventricular parameters are shown in **Tables 2**, **3**. The sports activities of the studied groups are presented in Supplementary Table 1.

All procedures performed in this study were under the 1964 Helsinki Declaration and its later amendments or comparable ethical standards. Ethics approval was obtained from the Central Ethics Committee of Hungary, and all participants provided informed consent.

### Image Acquisition and Analysis

The CMR examinations were performed with 1.5 T MRI scanners (Achieva, Philips Medical System, Eindhoven, the Netherlands and Magnetom Aera, Siemens Healthineers, Erlangen, Germany). Functional imaging was performed with balanced steady-state free precession cine sequences in 2-, 3-, and 4-chamber long-axis views and breath-hold short-axis views from base to apex with complete coverage of the LV and RV according to current recommendations (11, 12). The scan parameters acquired with the Philips Achieva and with the Siemens Aera scanners were the following: repetition time: 2.7 and 2.5 ms respectively, echo time: 1.3 and 1.15 ms, respectively, flip angle: 60° and 58°, respectively, spatial resolution: 1.5 × 1.5 mm in both scanners, temporal resolution: 25 frames per cardiac cycle in both scanners. The slice thickness was 8 mm with no interslice gap, the field of view 350 mm on average adapted to body size. A contrast agent was not administered.

**Figure 1:**
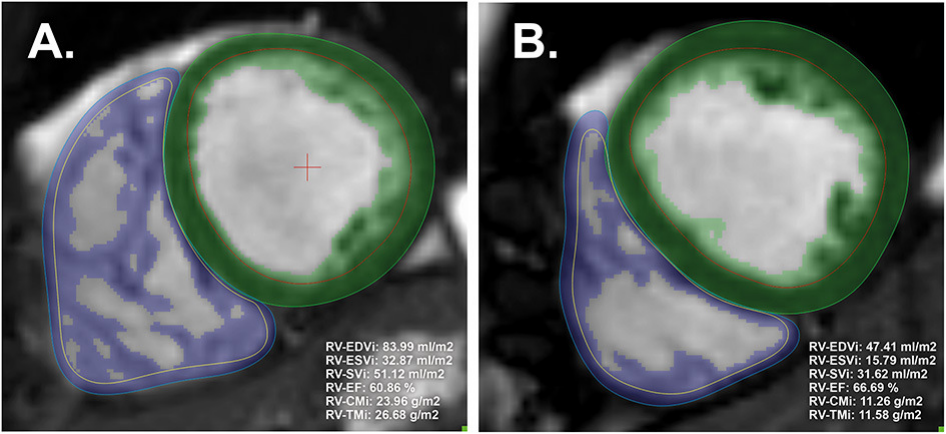
Representative image analysis with threshold-based papillary and trabeculated muscle quantification software and the calculated right ventricular parameters of male **(A)** and female **(B)** participants. The green and blue areas represent the myocardial mass, including the left and right ventricles' endocardial trabeculation (included in the endocardial contours). The threshold was set to the default (50%). CMi, end-diastolic compacted myocardial mass index; EDVi, end-diastolic volume index; EF, ejection fraction; ESVi, end-systolic volume index; LV, left ventricle; RV, right ventricle; SVi, stroke volume index; TMi, end-diastolic trabeculated myocardial mass index.

Medis Suite version 3.0 was used for the segmentation (Medis Medical Imaging Systems, Leiden, the Netherlands). Automatic tracing with manual correction of the endo- and epicardial contours of the LV and RV was performed by two observers (ASz with 10 years of experience and ARK with 5 years of experience). Inter-and-intraobserver agreement values are presented in Table 1. The LV and RV volumetric and functional values and the myocardial mass values were calculated with the MassK module of the software. This module performs a threshold-based papillary muscle and trabeculated myocardium quantification analysis that differentiates endocardial trabeculation from the blood pool based on the differing signal intensities of the blood and myocardium. Each voxel within the epicardial contour was classified as either blood or myocardium according to the chosen threshold, which was set to 50% (Figure 1) (9). Manual correction of the threshold was not applied. Voxels classified as myocardium within the endocardial contour were calculated as trabeculated myocardial mass, while compacted myocardial mass was calculated as the difference between the total detected and trabeculated myocardial mass. The following LV and RV parameters were calculated: left and right ventricular end-diastolic volume (LV-EDV, RV-EDV), end-systolic volume (LV-ESV, RV-ESV), stroke volume (LV-SV, RV-SV), ejection fraction (LV-EF, RV-EF), end-diastolic compacted myocardial mass (LV-CM, RV-CM), and end-diastolic trabeculated myocardial mass (LV-TM, RV-TM). The parameters were indexed (i) to body surface area. The following additional ratios were generated to study the relationship of volumetric parameters and myocardial mass: RV-trabeculated myocardial mass-to-compact myocardial mass (RV-TMi/RV-CMi), RV-trabeculated myocardial mass-to-end-diastolic volume (RV-TMi/RV-EDVi), and RV-compacted myocardial mass-to-end-diastolic volume (RV-CMi/RV-EDVi).

**Table 1:** Intraclass correlation coefficient (ICC) of the measured left and right ventricular parameters to describe inter-and intraobserver agreement.

	**Interobserver agreement (ICC)**	**Intraobserver agreement (ICC)**
LV-EDVi	0.98 (0.86-0.99)	0.99 (0.99-0.99)
LV-ESVi	0.96 (0.90-0.98)	0.95 (0.90-0.98)
LV-SVi	0.95 (0.85-0.98)	0.99 (0.98-0.99)
LV-EF	0.95 (0.89-0.98)	0.98 (0.95-0.99)
LV-TMi	0.99 (0.95-0.99)	0.98 (0.95-0.99)
LV-CMi	0.99 (0.98-0.99)	0.99 (0.98-0.99)
RV-EDVi	0.99 (0.97-0.99)	0.99 (0.98-0.99)
RV-ESVi	0.96 (0.93-0.99)	0.95 (0.87-0.97)
RV-SVi	0.99 (0.97-0.99)	0.99 (0.98-0.99)
RV-EF	0.98 (0.95-0.99)	0.92 (0.82-0.97)
RV-CMi	0.98 (0.95-0.99)	0.83 (0.62-0.92)
RV-TMi	0.99 (0.97-0.99)	0.98 (0.96-0.99)

*CMi, end-diastolic compacted myocardial mass index; EDVi, end-diastolic volume index; EF, ejection fraction; ESVi, end-systolic volume index; LV, left ventricle; RV, right ventricle; SVi, stroke volume index; TMi, end-diastolic trabeculated myocardial mass index*.

### Statistical Analysis

The intra-and interobserver agreement of the two observers was tested using the intraclass correlation coefficient (ICC). An ICC <0.4, between 0.4 and 0.75, and >0.75 indicates poor, fair to good, and excellent interobserver agreement. The Shapiro-Wilk test was used to assess the normality of each distribution. Normally distributed continuous variables are presented as the mean and standard deviation (SD), while median and interquartile range (IQR) is shown in the case of non-normal distribution. An unpaired Student's *t*-test or the Mann-Whitney U-test was used to compare male and female groups appropriately. The Pearson or Spearman test was used for correlation analysis as appropriate. Multiple linear regression analysis was applied to determine independent predictors for RV-compacted and RV-trabeculated myocardial mass. A *p*-value < 0.05 was considered statistically significant. IBM SPSS Statistics (Version 25.0, Armonk, NY) was used for calculations.

## Results

The inter- and intraobserver agreement of the two observers was tested on 25 randomly selected participants. All of the measured parameters showed excellent inter-and intraobserver agreement.

Studying the LV and RV parameters in the total population, we found significant differences between males and females: both LV and RV EDVi, ESVi, CMi, and TMi values were significantly larger, while the EF was significantly lower in males (Tables 2, 3).

**Table 2:** Baseline demographic and left ventricular characteristics of the studied male and female participants.

	**Male**	**Female**	***P***
Number of participants	100	100	–
Age	39.5 (29.3, 49.8)	40.0 (29.2, 49.5)	0.833
BSA (m^2^)	2.1 (2.0, 2.2)	1.7 (1.6, 1.8)	**<0.0001**
BMI (kg/m^2^)	25.5 (24.1, 27.5)	22.2 (20.3, 24.3)	**<0.0001**
LV-EDVi (ml/m^2^)	69.5 ± 10.8	64.4 ± 8.5	**0.0003**
LV-ESVi (ml/m2)	23.0 ± 5.3	19.3 ± 4.1	**<0.0001**
LV-SVi (ml/m^2^)	46.5 ± 7.9	45.1 ± 6.2	0.164
LV-EF (%)	67.0 ± 5.6	70.1 ± 4.4	**<0.0001**
LV-CMi (g/m^2^)	51.2 ± 8.0	40.5 ± 5.6	**<0.0001**
LV-TMi (g/m2)	22.8 ± 4.4	18.2 ± 3.3	**<0.0001**

*For the comparison between sexes, Mann-Whitney U-test was used in age, BSA, and BMI; otherwise, Student's t-test was used*.

*BMI, body mass index; BSA, body surface area; CMi, end-diastolic compacted myocardial mass index; EDVi, end-diastolic volume index; EF, ejection fraction; ESVi, end-systolic volume index; LV, left ventricle; SVi, stroke volume index; TMi, end-diastolic trabeculated myocardial mass index*.

*Bold values are statistically significant (p < 0.05)*.

**Table 3:** Right ventricular volumetric, functional, and myocardial mass values and the studied myocardial mass-to-volume ratios of the total population and the different age groups stratified by sex; and its comparison between sexes.

	**Group**		**Male**	**Female**	***P***
RV- EDVi (ml/m^2^)	Total population	Mean ± SD	70.3 ± 14.1	61.3 ± 9.4	**<0.0001**
	A	Mean ± SD	74.8 ± 11.8	66.5 ± 8.5	**0.006**
	B	Mean ± SD	71.5 ± 12.4	61.8 ± 8.5	**0.02**
	C	Mean ± SD	73.2 ± 13.4	61.9 ± 9.1	**0.001**
	D	Mean ± SD	61.7 ± 15.6	55.1 ± 8.5	0.068
RV-ESVi (ml/m^2^)	Total population	Mean ± SD	27.1 ± 6.0	21.2 ± 4.8	**<0.0001**
	A	Mean ± SD	29.3 ± 5.0	24.7 ± 4.4	**0.001**
	B	Mean ± SD	27.9 ± 6.2	21.4 ± 4.2	**<0.0001**
	C	Mean ± SD	27.6 ± 5.8	21.1 ± 15.5	**<0.0001**
	D	Mean ± SD	23.4 ± 5.7	17.6 ± 3.8	**<0.0001**
RV-SVi (ml/m^2^)	Total population	Mean ± SD	43.2 ± 9.8	40.1 ± 6.4	**0.007**
	A	Mean ± SD	45.6 ± 8.0	41.9 ± 6.4	0.086
	B	Mean ± SD	43.5 ± 8.9	40.4 ± 5.8	0.142
	C	Mean ± SD	45.6 ± 9.2	40.4 ± 6.7	**0.029**
	D	Mean ± SD	38.3 ± 11.4	37.5 ± 6.2	0.77
RV-EF (%)	Total population	Mean ± SD	61.4 ± 4.8	65.5 ± 5.1	**<0.0001**
	A	Mean ± SD	60.8 ± 3.4	63.2 ± 4.7	**0.048**
	B	Mean ± SD	60.8 ± 5.7	65.4 ± 4.4	**0.002**
	C	Mean ± SD	62.1 ± 4.9	65.3 ± 5.4	**0.034**
	D	Mean ± SD	62.0 ± 5.0	68.2 ± 4.8	**<0.0001**
RV-CMi (g/m^2^)	Total population	Mean ± SD	16.4 (15.0, 19.1)	13.4(12.0, 14.8)	**<0.0001**
	A	Mean ± SD	16.8 ± 3.4	13.4 (12.1, 15.3)	**0.002**
	B	Mean ± SD	16.6 ± 2.7	13.7 ± 1.8	**<0.0001**
	C	Mean ± SD	18.0 ± 3.5	13.4 (12.5, 15.3)	**<0.0001**
	D	Mean ± SD	17.0 ± 3.1	13.4 ± 2.0	**<0.0001**
RV-TMi (g/m^2^)	Total population	Mean ± SD	20.1 ± 4.2	15.3 ± 3.3	**<0.0001**
	A	Mean ± SD	19.9 ± 3.7	17.1 ± 3.6	**0.009**
	B	Mean ± SD	19.3 ± 3.8	14.9 ± 3.0	**<0.0001**
	C	Mean ± SD	21.8 ± 5.2	13.9 (11.9, 19.3)	**<0.0001**
	D	Mean ± SD	19.5 ± 3.6	14.3 ± 2.8	**<0.0001**
RV-TMi/RV-CMi	Total population	Mean ± SD	1.21 ± 0.29	1.12 ± 0.27	**0.029**
	A	Mean ± SD	1.23 ± 0.30	1.24 ± 0.30	0.889
	B	Mean ± SD	1.18 ± 0.25	1.10 ± 0.25	0.217
	C	Mean ± SD	1.24 ± 0.35	1.05 ± 0.24	**0.032**
	D	Mean ± SD	1.18 ± 0.26	1.09 ± 0.24	0.181
RV-TMi/RV-EDVi	Total population	Mean ± SD	0.28 (0.24, 0.33)	0.24 (0.21, 0.28)	**<0.0001**
	A	Mean ± SD	0.27 (0.22, 0.31)	0.25 (0.22, 0.30)	0.527
	B	Mean ± SD	0.27 (0.24, 0.30)	0.24 (0.21, 0.28)	0.062
	C	Mean ± SD	0.28 (0.25, 0.33)	0.23 (0.21, 0.26)	**0.001**
	D	Mean ± SD	0.32 (0.28, 0.39)	0.26 (0.23, 0.30)	**0.008**
RV-CMi/RV-EDVi	Total population	Mean ± SD	0.24 (0.21, 0.28)	0.22 (0.20, 0.25)	**0.018**
	A	Mean ± SD	0.22 (0.20, 0.24)	0.21 (0.19, 0.24)	0.445
	B	Mean ± SD	0.23 (0.21, 0.26)	0.22 (0.19, 0.25)	0.75
	C	Mean ± SD	0.24 (0.22, 0.30)	0.23 (0.21, 0.25)	0.139
	D	Mean ± SD	0.27 (0.24, 0.31)	0.25 (0.22, 0.28)	**0.025**

*For the comparison between sexes, Mann-Whitney U-test was used in case of the following parameters: RV-CMi in the total population and Group A and C in females, in the total population and Group C in males; RV-TMi in Group C in females; RV-TMi/RV-EDVi in every group in both males and females; RV-CMi/RV-EDVi in every group in both males and females. Otherwise, Student's t-test was used*.

*CMi, end-diastolic compacted myocardial mass index; EDVi, end-diastolic volume index; EF, ejection fraction; ESVi, end-systolic volume index; RV, right ventricle; RV-CMi/RV-EDVi, right ventricular end-diastolic compacted myocardial mass index-to-right ventricular end-diastolic volume index ratio; RV-TMi/RV-CMi, right ventricular end-diastolic trabeculated myocardial mass index-to-right ventricular end-diastolic compacted myocardial mass index ratio; RV-TMi/RV-EDVi, right ventricular end-diastolic trabeculated myocardial mass index-to-right ventricular end-diastolic volume index ratio; SVi, stroke volume index; TMi, end-diastolic trabeculated myocardial mass index*.

*Bold values are statistically significant (p < 0.05)*.

We further described the sex-related differences in each age group (Table 3). The volumetric, functional, and myocardial mass values significantly differed between males and females in each age group, similar to the total population. However, the RV-EDVi value was not significantly different between the sexes in Group D. The RV-SVi value was larger in males; still, the difference was significant only in Group C (Table 3).

Regarding sex-related differences of the studied ratios, the RV-TMi/RV-CMi ratio was comparable in the youngest age group between males and females, but it was higher in males than in females in Group B, Group C, and D and was significantly different between sexes in Group C. The RV-CMi/RV-EDVi and RV-TMi/RV-EDVi ratios were larger in males. The former was significantly different in Group C, and the latter was significant in Group C and Group (Table 3).

**Figure 2:**
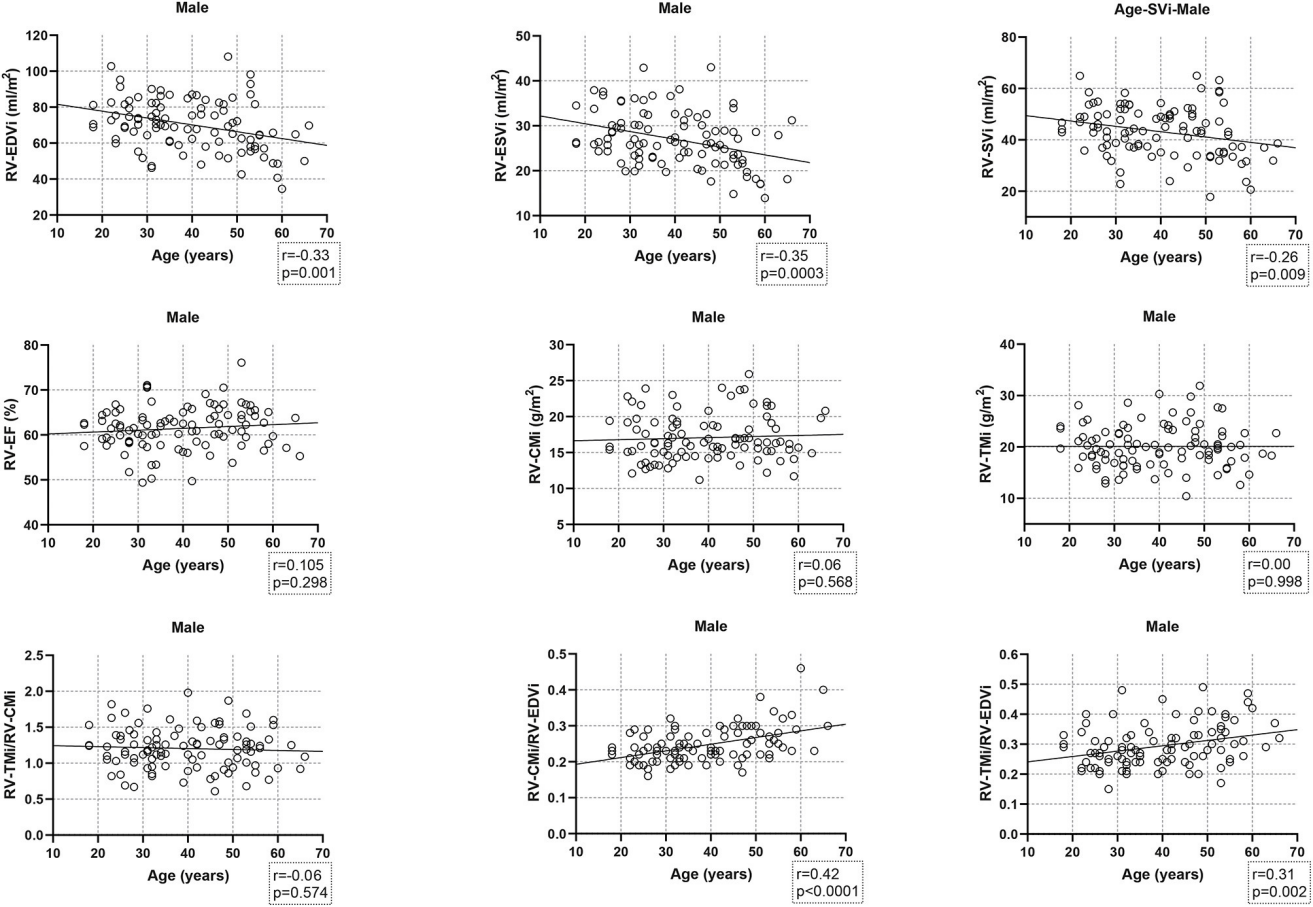
Correlation between age and right ventricular volumetric, functional, and myocardial mass values, and the studied ratios in males. CMi, end-diastolic compacted myocardial mass index; EDVi, end-diastolic volume index; EF, ejection fraction; ESVi, end-systolic volume index; RV, right ventricle; SVi, stroke volume index; TMi, end-diastolic trabeculated myocardial mass index.

**Figure 3:**
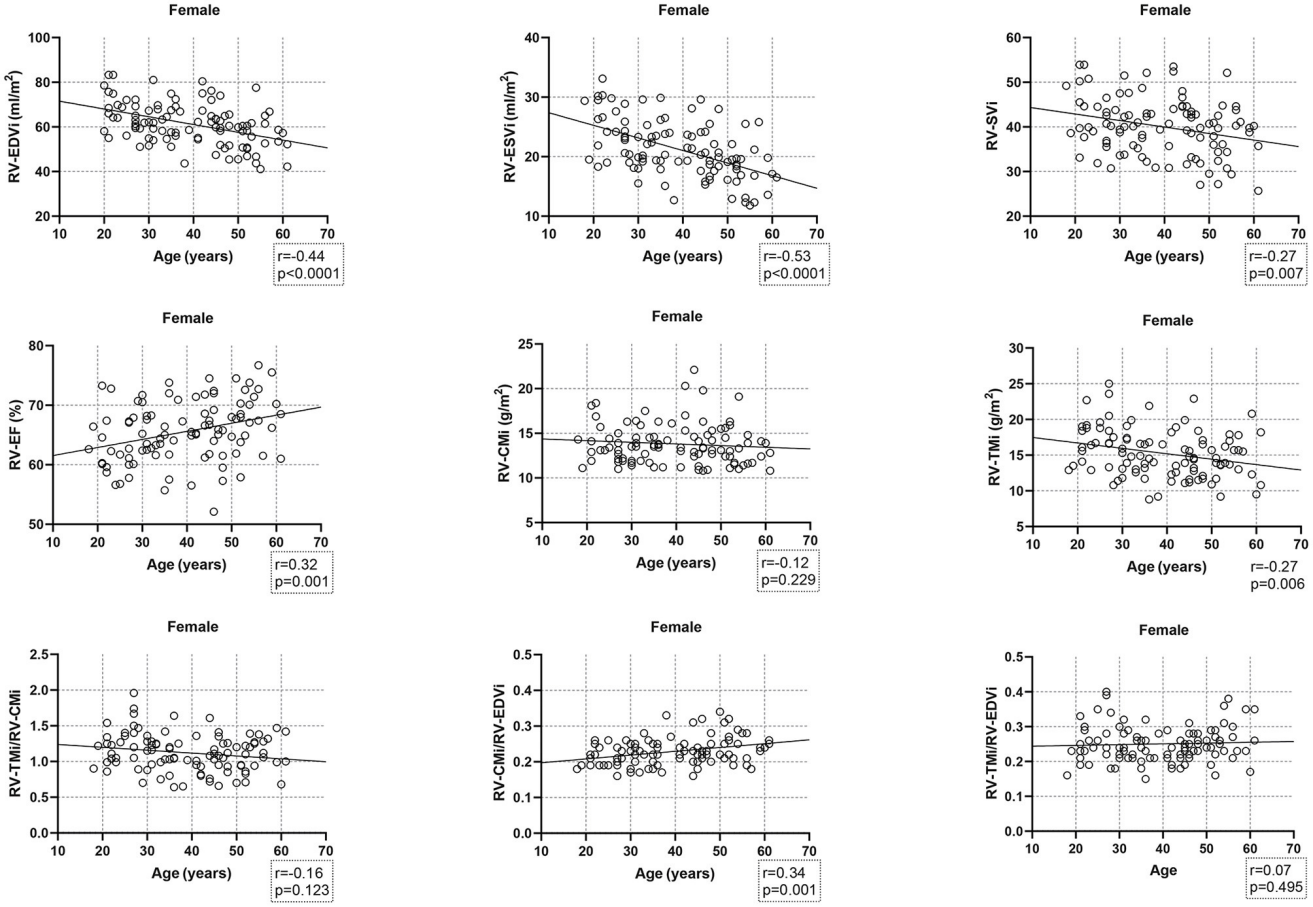
Correlation between age and right ventricular volumetric, functional, and myocardial mass values, and the studied ratios in females. CMi, end-diastolic compacted myocardial mass index; EDVi, end-diastolic volume index; EF, ejection fraction; ESVi, end-systolic volume index; RV, right ventricle; SVi, stroke volume index; TMi, end-diastolic trabeculated myocardial mass index.

Age-related changes in the studied parameters were as follows (Figures 2, 3). The RV-EDVi, RV-ESVi, and RV-SVi showed a significant negative correlation with age in both sexes. The RV-EF was positively correlated with age in females but not in males. The RV-CMi value was not associated with age in either of the sexes, while the RV-TMi value negatively correlated with age in females.

The RV-TMi/RV-CMi did not correlate with age in either males or females, while the RV-CMi/RV-EDVi ratio positively correlated with age in both sexes, and the RV-TMi/RV-EDVi ratio showed a positive correlation only in males.

Linear relationships were studied between RV-TMi, RV-CMi, RV volumetric and functional parameters, and LV myocardial mass values (Table 4). RV-TMi was positively correlated with RV-ESVi, RV-EDVi, RV-CMi, LV-TMi, and LV-CMi in both sexes and negatively correlated with age and RV-EF in females. In both sexes, the RV-CMi significantly correlated with the RV-EDVi, RV-TMi, LV-CMi, and LV-TMi; however, it correlated significantly with the RV-ESVi and RV-EF only in males.

**Table 4:** Correlation between right ventricular compacted and trabeculated myocardial mass values and right ventricular volumetric and functional and left ventricular myocardial mass values.

			**RV-ESVi**	**RV-EDVi**	**RV-EF**	**RV-TMi**	**RV-CMi**	**LV-TMi**	**LV-CMi**
RV-TMi	Male	r	0.32	0.35	0.03	1.00	0.27	0.35	0.38
		*P*	**0.001**	**0.0004**	0.799	–	**0.007**	**0.0003**	**0.0001**
	Female	r	0.532	0.45	−0.37	1.00	0.24	0.32	0.36
		*P*	**<0.0001**	**<0.0001**	**0.0002**	–	**0.018**	**0.001**	**0.0003**
RV-CMi	Male	r	0.39	0.58	0.24	0.27	1.00	0.27	0.46
		*P*	**<0.0001**	**<0.0001**	**0.015**	**0.007**	–	**0.006**	**<0.0001**
	Female	r	0.30	0.48	0.04	0.24	1.00	0.22	0.47
		*P*	0.002	**<0.0001**	0.686	**0.018**	–	**0.026**	**<0.0001**

*Spearman test was used to correlate RV-CMi to the other parameters; otherwise, Pearson test was used*.

*CMi, end-diastolic compacted myocardial mass index; EDVi, end-diastolic volume index; EF, ejection fraction; ESVi, end-systolic volume index; LV, left ventricle; RV, right ventricle; TMi, end-diastolic trabeculated myocardial mass index*.

*Bold values are statistically significant (p < 0.05)*.

Multiple regression analysis was performed on the total population to find independent predictors of RV-TMi and RV-CMi (Table 5). RV-TMi was independently associated with sex, RV-ESVi, LV-TMi, and LV-CMi, with a cumulative *R*-value of 0.701 (*p* < 0.0001). Independent predictors of RV-CMi were age, sex, RV-EDVi, and LV-CMi, with a cumulative *R*-value of 0.728 (*p* < 0.0001).

**Table 5:** Multivariate linear regression analysis and the independent predictors of right ventricular compacted and trabeculated myocardial mass.

	**RV-TMi**			**RV-CMi**	
**Covariate**	**β**	***P***	**Covariate**	**β**	***P***
Age	0.002	0.978	Age	0.175	**0.001**
Sex	0.152	**0.030**	Sex	0.219	**0.001**
RV-EDVi	−0.061	0.552	RV-EDVi	0.490	**<0.0001**
RV-ESVi	0.264	**<0.0001**	RV-ESVi	−0.159	0.106
RV-SVi	−0.048	0.475	RV-SVi	0.214	0.102
RV-EF	−0.057	0.425	RV-EF	0.082	0.134
RV-CMi	−0.006	0.934	RV-TMi	0.014	0.825
LV-CMi	0.225	**0.002**	LV-CMi	0.186	**0.014**
LV-TMi	0.253	**<0.0001**	LV-TMi	0.086	0.152
Cumulative R	0.701		Cumulative R	0.728	
Standard error	3.24		Standard error	2.22	
Cumulative P	**<0.0001**		Cumulative P	**<0.0001**	

*CMi, end-diastolic compacted myocardial mass index; EDVi, end-diastolic volume index; EF, ejection fraction; ESVi, end-systolic volume index; LV, left ventricle; RV, right ventricle; SVi, stroke volume index; TMi, end-diastolic trabeculated myocardial mass index*.

*Bold values are statistically significant (p < 0.05)*.

## Discussion

In this study, we assessed the characteristics of RV in different age groups stratified by sex. We described smaller RV volumes and higher RV-EF, while the RV compact myocardial mass values were similar to those published (2, 13). During conventional contour-based post-processing techniques, trabeculae are included in the endocardial contours and are calculated as blood volume; however, they contribute approximately 50% to the RV myocardial mass (14). The threshold-based software used in our study is a highly reproducible technique with an excellent inter-and intraobserver agreement that enables us to measure both total and trabeculated myocardial mass values accurately. However, volumetric parameters are significantly lower compared to conventional techniques (9, 14). Average RV total myocardial mass values have already been established, but the reference ranges of compacted and trabeculated RV myocardial mass are less well-studied. We found only one publication that quantified RV trabeculation; however, they used manual contouring and defined myocardium volumes (15). After converting these RV trabeculated myocardium values from volume to mass, we found that Andre et al. described higher RV trabeculated mass values than those in our results. It might be due to the different post-processing techniques; namely, they used manual contouring of the endo- and epicardial borders compared to the threshold-based software applied in our study (15, 16). The use of normal ranges of RV compacted and trabeculated myocardial mass might have diagnostic, differential diagnostic, and prognostic relevance in cases where RV hypertrabeculation or RV hypertrophy is present (5–8). Furthermore, our study's myocardial mass ratios and the myocardial mass-to-volume ratios might also help in the differential diagnosis of physiological (e.g., athletes' heart, pregnancy) and pathological conditions resulting in volume or pressure overload of the RV. Our study's presented age- and sex-specific values could be used as standard reference ranges for this threshold-based software with the same threshold setting as in our study.

Assessing the sex-related differences in the studied RV parameters, we found higher compacted and trabeculated myocardial mass values, higher volumetric parameters, and smaller RV-EF in males. These were consistent with the literature and could be explained by biometric and hormonal differences between sexes (1, 15, 17). Previous studies described the age-related decrease in RV functional parameters, which is in line with our results; however, there are inconsistent data about the changes in RV myocardial mass (18, 19). Kawut et al. and Maceira et al. described a significant decrease in RV myocardial mass over time, although they studied wider age ranges with older participants than ours (1, 18). Sandstede et al. studied similar age groups and found unchanged RV mass values with age, similar to our compacted myocardial mass results (19). The relatively stable RV-compacted mass values might be explained by the age-related changes in the myocardium characterized by the loss of myocytes with an increase in cell volume, resulting in compensatory hypertrophy. This mechanism could be the cause of unchanged compacted myocardial mass values (20).

Regarding the trabeculated myocardial mass, we found a slight decrease with age in females, and this negative age dependency is in line with Andre's results, although the mechanism is unknown (15, 21). In contrast to previous results, the RV trabeculated mass was not different between the age groups and was higher in older males than in females of the same age in our study (15). A possible explanation for these altering results could be the abovementioned difference in post-processing techniques. We did not find information about the sex- or age-related factors and mechanisms influencing RV trabeculation; however, we hypothesize that physical activity might play a role. It is known that excessive LV and RV trabeculation can be found in athletes as part of the process of adaptation to intense physical activity (22, 23). Woodbridge et al. studied the relationship between physical activity and the extent of LV trabeculation in a community-based cohort and found no linear relationship (24). They propose that there might be a threshold in physical activity that must be exceeded to manifest an increase in LV trabeculation. It is also known that there is a slight difference between LV and RV sport adaptation, as the right ventricle shows slightly stronger morphological changes (25). Thus, we hypothesize that RV trabeculation develops more easily than LV trabeculation and might be more sensitive to physical activity that does not exceed the training hours of competitive athletes and does not result in changes in RV volumes (10). A possible mechanism is that regular exercise increases the circulating level of testosterone, which might lead to muscle growth through increased protein synthesis and decreased protein degradation in males (26). This hypothesis might explain the comparable RV trabeculated myocardial mass value between the studied age groups in males; however, the cause of the slight age-related decrease in RV-TMi in females remains unanswered.

The RV-EF was positively correlated with age in females but not in males. The data are controversial about the age-related changes in the RV-EF: Petersen et al. found an age-related increase in females only, which is similar to our results, while Maceira et al. described an increase in the RV-EF in males as well. However, the RV-EF did not change significantly over time in either males or females in Sandstede's study (2, 18, 19).

The RV trabeculated myocardial mass-to-compact myocardial mass ratio did not show sex-related differences and was not correlated with age. To the best of our knowledge, there is no information about the RV trabeculated myocardial mass-to-compact myocardial mass ratio in healthy male and female populations. However, previous studies found no significant association between sex and non-compacted-to-compacted myocardial thickness of the LV (27, 28). The results of Gregor et al. might also strengthen this; namely, there is no significant difference between the LV trabeculated myocardial mass-to-compact myocardial mass ratio in males and females in either younger or older age groups measured with threshold-based software (29). However, we need to mention that physiological mechanisms affecting RV and LV trabeculation might be different.

Different myocardial mass-to-volume ratios have been previously studied in the left ventricle but not in the right ventricle. Czimbalmos et al. described the high diagnostic accuracy of LV muscle-to-volume ratios in the differential diagnosis of hypertrophic cardiomyopathy and athletes' hearts (30). The RV-TMi/RV-EDVi and RV-CMi/RV-RDVi ratios in our study correlated positively with age. They might help identify the early stages of different pathological conditions that affect the RV.

In studying the relationship between RV and LV myocardial mass values and RV volumetric parameters, we found that compacted RV myocardial mass correlated with trabeculated RV myocardial mass and vice versa. Furthermore, both compacted and trabeculated RV masses correlated with LV compacted and trabeculated myocardial masses, and LV trabeculation was an independent predictor of RV trabeculation. We did not find previous studies about the connection between either RV or LV compacted and trabeculated myocardial masses. Some researchers suggest that left and right ventricular myocardial mass is genetically determined; however, the degree of trabeculation might vary between the ventricles due to the different physiological mechanisms and stimulating factors (15). RV volumetric parameters showed a strong relationship with compacted and trabeculated RV myocardial mass values. Moreover, the RV-ESVi was an independent predictor of RV trabeculation, while the RV-EDVi was an independent predictor of RV-compacted myocardium. Previous studies conducted in a healthy population, pregnant women, patients with chronic anemia, patients with heart failure, and high-level athletes suggest that LV trabeculation is strongly related to LV volumes. Furthermore, excessive LV trabeculation is a normal response to increased cardiac preload and ventricular volume (21, 27, 31–33). We did not find previous publications about the impact of RV volumes on RV trabeculation, but the relationship might not be surprising. RV-compacted myocardial mass was previously described to correlate with RV volumes positively, LV compacted myocardial mass, and pulmonary artery pressure, which is in line with our results (34).

To summarize, the RV-compacted myocardial mass remained stable over time in both sexes, but the RV trabeculated myocardial mass decreased with age in females. RV trabeculated myocardial mass correlated significantly with RV-compacted myocardial mass and vice versa. Furthermore, LV compacted, and trabeculated myocardial mass values and RV volumetric parameters were independent predictors of RV-compacted and trabeculated myocardial mass. The age- and sex-related characteristics of RV compacted and trabeculated myocardial masses described in a healthy population have not been previously studied; however, this additional information might help differentiate conditions with RV hypertrophy or hypertrabeculation.

We need to mention the limitations of this study. It was conducted on a large cohort; however, the subgroups were relatively small after dividing by age. This study did not cover elderly populations as we needed to exclude these participants due to cardiovascular or other systemic diseases (mainly hypertension). The CMR cine images were acquired using two different CMR scanners, and although the scan parameters were similar, this might be considered a limitation. We need to mention a few limitations regarding the threshold-based software as well. The current ejection fraction and volume quantification use a stack of thick short-axis slices, and 8 mm is common for Z-direction spatial resolution. In the meantime, trabeculae and papillary muscles do not cross the slice in an exactly perpendicular fashion, which creates partial volume effects. Depending on the actual path of the trabeculae, this will influence the threshold-based quantification (9, 16). The reported values in this study were measured with the threshold set to 50%. However, altering the threshold might change the measured myocardial mass values. Papillary muscles were counted as trabeculation in our study because of the nature of this technique. Previous studies on LV trabeculation excluded the papillary muscles from trabeculation, making the comparison of the different results more challenging. However, researchers suggest that the segmentation of papillary muscles does not make a significant difference (15).

## Data Availability Statement

The raw data supporting the conclusions of this article will be made available by the authors, without undue reservation.

## Ethics Statement

Ethical review and approval was not required for the study on human participants in accordance with the local legislation and institutional requirements. The patients/participants provided their written informed consent to participate in this study.

## Author Contributions

AK: methodology, formal analysis, investigation, resources, data curation, writing—original draft, and visualization. ZG: data curation. ÁF: data curation. LS: writing—review and editing. ZD: writing—review and editing. BM: supervision. HV: writing—review and editing and supervision. AS: conceptualization, methodology, investigation, resources, writing—review and editing, supervision, and project administration. All authors contributed to the article and approved the submitted version.

## Funding

This research was financed by the Thematic Excellence Programme (Tématerületi Kiválósági Program, 2020-4.1.1.-TKP2020) of the Ministry for Innovation and Technology in Hungary within the framework of the Therapeutic Development and Bioimaging Programs of Semmelweis University; by the Development of Scientific Workshops of Medical, Health Sciences and Pharmaceutical Education (Project identification number: EFOP-3.6.3-VEKOP-16-2017-00009); and by the Ministry of Innovation and Technology NRDI Office within the framework of the Artificial Intelligence National Laboratory Program. Project no. NVKP_16-1–2016-0017 (National Heart Program) has been implemented with the support provided by the National Research, Development, and Innovation Fund of Hungary, financed under the NVKP_16 funding scheme.

## Conflict of Interest

The authors declare that the research was conducted in the absence of any commercial or financial relationships that could be construed as a potential conflict of interest.

## Publisher's Note

All claims expressed in this article are solely those of the authors and do not necessarily represent those of their affiliated organizations, or those of the publisher, the editors and the reviewers. Any product that may be evaluated in this article, or claim that may be made by its manufacturer, is not guaranteed or endorsed by the publisher.
