# Supplementary material for: Age- and Sex-Specific Characteristics of Right Ventricular Compacted and Non-compacted Myocardium by Cardiac Magnetic Resonance
Source: Front Cardiovasc Med. 2021 Dec 7;8:781393. doi: 10.3389/fcvm.2021.781393 (PMC8688768; doi:10.3389/fcvm.2021.781393)
Supplement: Supplementary file 1 [file Table_1.docx]

**Supplementary** **Table 1.** Average sports activity of the studied groups. Bold values indicate staticially significant values p<0.05).

# p< 0.05 vs. Group B; & p< 0.05 vs. Group C

|  |  | **Group A** | **Group B** | **Group C** | **Group D** | ***P*** |
| --- | --- | --- | --- | --- | --- | --- |
| **Sports activity (hours/week)** | **male** | 2.2±1.5 | 3.3±1.6 | 3.1±2.2 | 3.9±1.4 | 0.057 |
|  | **female** | 2.7±1.1 | 3.7±2.8 ^&^ | 1.8±2.3 ^#^ | 2.5±1.8 | **0.04** |
